# Fetoplacental Discrepancy with Normal Karyotype in Amniotic Fluid and Two Different Cell Lines in Placenta

**DOI:** 10.1155/2013/951710

**Published:** 2013-06-13

**Authors:** Veronica Ortega, Christina Mendiola, Eric Williamson, Kenneth Higby, Gopalrao V. N. Velagaleti

**Affiliations:** ^1^Department of Pathology, University of Texas Health Science Center, San Antonio, TX 78229, USA; ^2^Center for Maternal and Fetal Care, San Antonio, TX 78229, USA

## Abstract

We present a case of fetoplacental discrepancy in a second-trimester fetus with normal karyotype in amniotic fluid and two different Robertsonian translocations in placenta. A 41-year-old woman of Middle-Eastern origin, gravida 2, para 1, underwent amniocentesis at 16-week gestation because of advanced maternal age. Amniotic fluid karyotype showed a normal 46,XX karyotype with a homozygous inv(9). Parental chromosome analysis showed both parents to be carriers of inv(9) and the parents are not consanguineous. Fetal ultrasound was normal. The mother presented to the clinic 4 weeks later with intrauterine fetal demise. Chromosome analysis from the placenta showed two different cell lines: a balanced (15;21) Roberstonian translocation in 11 cells and an unbalanced (21;21) Robertsonian translocation in 9 cells. The karyotype was interpreted as mos 45,XX,inv(9)(p11q13)x2,der(15;21)(q10;q10)[11]/46,XX,inv(9)(p11q13)x2,+21,der(21;21)(q10;q10). Mother was a carrier for the Cystic Fibrosis (delta F508), Factor V Leiden mutations, HbD-Los Angeles and HbQ-India variants. She also had a sibling with term stillbirth. Her husband's history was unremarkable. Our case appears to be another example of confined placental mosaicism (CPM) with normal fetal karyotype. However, we could not confirm the possibility that CPM contributed to the IUFD in our case given the complex medical history of the mother.

## 1. Introduction

Intrauterine fetal death (IUFD) is a major problem in obstetrics. The overall IUFD rates have been estimated to be approximately 1% [1]. In pregnancy, a successful outcome is highly dependent on adequate placental development with low-resistance fetomaternal circulation [2]. Abnormal placentation in pregnancy could be due to several factors including confined placental mosaicism (CPM) and increased maternal tendency to venous thrombosis. Karyotype disparities between cytotrophoblast and fetal cells occur in 1-2% of cases [3]. In most of them, chromosomal abnormalities are confined to the placenta and may be associated with a poor perinatal outcome [4, 5]. Most such chromosome abnormalities involve aneuploidies, and autosomal trisomy being most common with structural mosaicism is rare and often difficult to interpret [6]. The phenomenon of “confined placental mosaicism (CPM)” is defined as tissue-specific mosaicism involving a cytogenetic abnormality limited to the placenta and absent in the fetus arising from an early mitotic error [7, 8]. Three types of CPM have been described. First, trisomic cytotrophoblast and diploid stroma; second, diploid cytotrophoblast and trisomic chorionic stroma; and third, trisomic cytotrophoblast and chorionic stroma. In all three types, the mosaicism is confined to the placenta and the fetus is normal [7]. Fetal growth retardation and death were shown to be associated with an increasing proportion of aneuploidic cells in the placenta and placental lineage, even when the chromosomal anomaly is exclusively confined to the placenta, suggesting a major influence for the placenta on abnormal fetal development [9–11]. Abnormal placentation early in pregnancy results in restricted blood flow to the placental-fetal unit. Increased maternal susceptibility to venous thrombosis has been associated with abnormal placentation [12]. Genetic risk factors for venous thrombosis, such as Factor V Leiden and prothrombin G20210A mutation, were implicated as causes of abnormal placentation [12, 13].

Here, we report an unusual case of fetoplacental discrepancy in terms of chromosome abnormality and other maternal genetic risk factors including Factor V Leiden and hemoglobin variants.

## 2. Case Report

A 37-year-old woman of Middle-Eastern origin, gravida 2, para 1, underwent amniocentesis at 16-week gestation because of advanced maternal age. The mother had a significant medical and family history. She is heterozygous for the ΔF508 mutation, Factor V Leiden mutation, and also the hemoglobin D-Los Angeles (HbD-Punjab) and hemoglobin Q-India (HbQ-India) variants. The father was 36 years old and had no significant past medical history and is not a carrier of either ΔF508 or Factor V Leiden mutations. 

**Figure 1:**
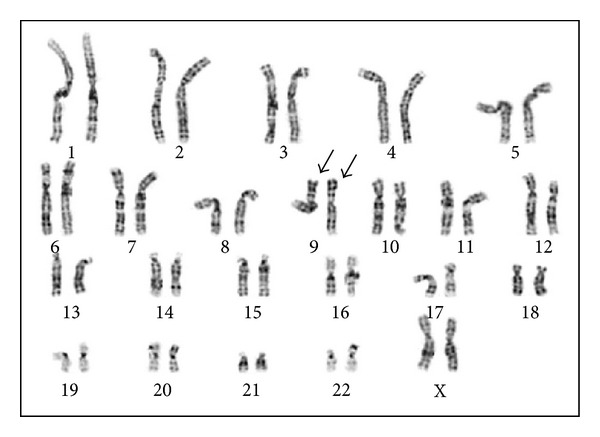
Karyotype from the amniotic fluid. The arrows point to the homozygous inv(9) chromosomes.

Prenatal aneuploidy testing showed a signal pattern consistent with normal female chromosomes, while amniotic fluid chromosome analysis confirmed a normal 46,XX karyotype. Interestingly, the karyotype also showed a homozygous inv(9) in all the analyzed cells (Figure 1). Fetal ultrasound was normal and did not show any unusual findings. Although the inv(9) is considered a polymorphic variant, due to the homozygous nature, parental chromosome studies were suggested. Both parents showed the inv(9) in their respective karyotypes, thus suggesting the biparental inheritance of the homozygous inv(9) in the fetus.

**Figure 2:**
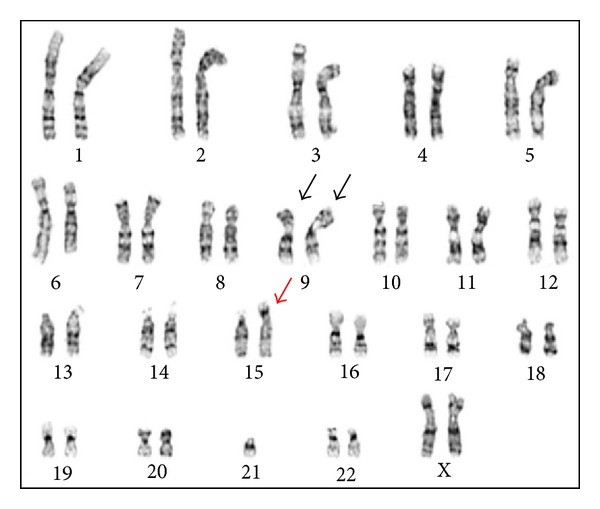
Karyotype from placenta showing the balanced 15;21 translocation (red arrow).

**Figure 3:**
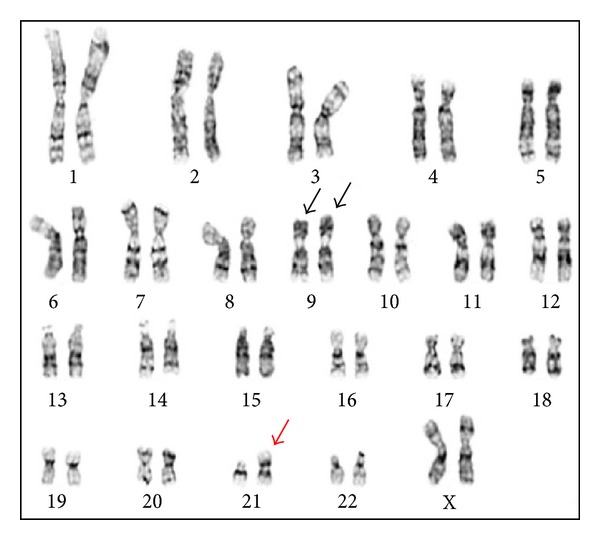
Karyotype from placenta showing the unbalanced 21;21 translocation (red arrow).

The mother presented to the clinic 4 weeks later with intrauterine fetal demise. Although cultures were initiated from fetal, cord, and placental tissues, growth was obtained only from placental tissue. Chromosome analysis showed two different cell lines: a balanced (15;21) Robertsonian translocation in 11 cells and an unbalanced (21;21) Robertsonian translocation in 9 cells. The karyotype was interpreted as mos 45,XX,inv(9)(p11q13)x2,der(15;21)(q10;q10)[11]/46,XX,inv(9)(p11q13)x2,+21,der(21;21)(q10;q10) [14] (Figures 2 and 3).

After extracting the DNA from the placental tissue culture sample, Factor V Leiden G1691A and prothrombin G20210A mutations were detected by TaqMan allelic discrimination method. The results showed that the placental tissue is heterozygous for the Factor V Leiden G1691A mutation while no G20210A mutations were detected in the prothrombin gene.

## 3. Discussion

Despite intensive research, etiology of intrauterine fetal demise remains unknown in about 25% of cases. Adequate placental development is essential for successful outcome in pregnancy. Chromosome abnormalities and genetic risk factors for thrombosis are major factors in abnormal placentation.

Discrepant results either between direct analysis and long-term culture of chorionic villi (CVS) or between CVS and amniocentesis occur in about 1% to 2% of cases [3]; however, such discrepant results rarely involve structural rearrangements [3, 11]. Mosaic trisomy 21 resulting from a structural rearrangement is rare and is only reported once in prenatal diagnosis [15]. In our case, although the amniotic fluid showed a normal karyotype, chromosome analysis from the placental tissue showed mosaicism with two structural rearrangements, including an unbalanced Robertsonian translocation leading to trisomy 21 and a balanced Robertsonian translocation. This is the first such case where the fetoplacental discrepancy involved mosaicism with one balanced and one unbalanced Robertsonian translocation. As has been reported, our case further strengthens the hypothesis that placenta has a major influence on the fetal development and chromosome abnormalities in placenta result in fetal growth retardation and fetal demise. 

Although it has been hypothesized that mothers or fetuses with an acquired or genetic predisposition may have abnormal thrombosis and infarction of the uteroplacental circulation that might result in adverse pregnancy complications including pregnancy loss and fetal growth restriction, the reports of retrospective studies are conflicting [2]. One study reported increased risk of fetal loss in Caucasian women with delimited Mediterranean area with Factor V Leiden mutations [16]. In this context, it is interesting that our patient is of Mediterranean origin and is heterozygous for the Factor V Leiden mutation. Several studies have suggested evaluation of Factor V Leiden mutations in women with unexplained fetal loss following exclusion of chromosomal abnormalities, infections, anatomic alterations, and endocrinological dysfunction [17–19]. In our case, the fetal loss occurred in the second trimester and a recent study indicated that pregnancy loss with Factor V Leiden mutations occurred throughout pregnancy in women with thrombophilia, although such loss is more frequent in late pregnancy in women with thrombophilia [19]. However, our patient did not have thrombophilia and the fetal loss occurred during second trimester. Similarly, fetal loss resulting from chromosome abnormalities occurs more frequently during first trimester pregnancy than late pregnancies [20]. In this context, our case is unique in that fetal loss occurred later in pregnancy and the etiology of this fetal loss is complicated due to presence of both chromosome abnormalities and significant maternal history of genetic predisposition.
